# miR-125b differentially impacts mineralization in dexamethasone and calcium-treated human mesenchymal stem cells

**DOI:** 10.1016/j.omtn.2024.102446

**Published:** 2025-01-03

**Authors:** Virginie Joris, Elizabeth R. Balmayor, Martijn van Griensven

**Affiliations:** 1 Department of Cell Biology-Inspired Tissue Engineering (cBITE), MERLN Institute for Technology-Inspired Regenerative Medicine, Maastricht University, Universiteitssingel 40, 6229 ER Maastricht, the Netherlands; 2 Experimental Orthopaedics and Trauma Surgery, Department of Orthopaedic, Trauma, and Reconstructive Surgery, RWTH Aachen University Hospital, Pauwelsstrasse 30, 52074 Aachen, Germany

**Keywords:** MT: Non-coding RNAs, dexamethasone, calcium, miR-125b, mineralization, molecular mechanisms, differential effect

## Abstract

Bone metabolism is highly regulated, and microRNAs (miRs) can contribute to this process. Among them, miR-125b is well known to enhance osteoporosis and reduce osteogenic differentiation of human mesenchymal stem cells (hMSCs). In this work, we aim to evaluate and understand how miR-125b modulates mineralization of hMSCs in two different *in vitro* models. Cells were cultured in dexamethasone or calcium medium and transfected with miR-125b mimic. Exposure to dexamethasone or calcium medium increased the mineralization of hMSCs and was associated with decreased miR-125b expression. Transfection of miR-125b mimic in dexamethasone-treated cells increased mineralization, while it decreased it in calcium-treated cells. Levels of osteogenic markers presented the same difference. We identified STAT3, p53, and RUNX2 as direct targets of miR-125b in hMSCs. While these targets remained identical in both treatments, their modulation after transfection was different. We showed that miR-125b mimicking differentially modulated the expression of the miR-199a/214 cluster, probably via STAT3/miR-199a/214 and p53/miR-214 pathways. In conclusion, miR-125b affinity for targets implicated in bone remodeling changed depending on the *in vitro* models used to induce mineralization and led to opposite physiological effects. This work shows the complexity of drugs such as dexamethasone and opens the door for new *in vitro* models of mineralization.

## Graphical abstract

**Figure:**
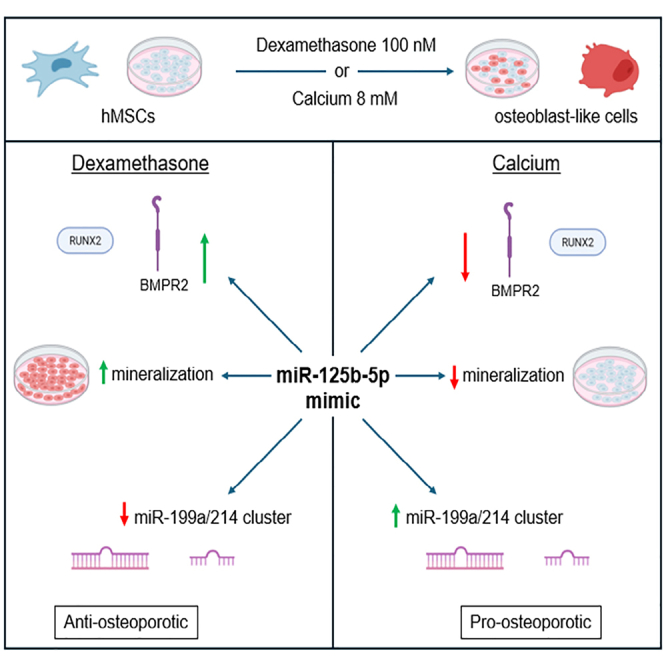

## Teaser

Joris and colleagues showed that miR-125b mimicking has the opposite effect on dexamethasone or calcium-induced mineralization of hMSCs. This is associated with differential regulation of osteogenic proteins and microRNAs. We highlighted the complexity of bone mechanisms and brought to light a new *in vitro* approach and new potential targets in bone formation.

## Introduction

Osteoporosis is a silent systemic skeletal disorder characterized by deregulations of bone homeostasis leading to the deterioration of bone tissue and bone fragility.^1^ Recent estimations showed that 33% of women and 20% of men are at risk of developing osteoporosis,^1^ making it a burdening health problem. Disruption of bone homeostasis during osteoporosis is happening through an increase in bone resorption, mediated by osteoclasts and a decrease in bone formation mediated by osteoblasts. While strategies targeting osteoclast activity have already shown promising results in the clinic to halt or reverse osteoporosis, stimulating osteoblast maturation to induce bone formation is a less-developed approach.^2^ Moreover, mesenchymal stem cells (MSCs) appear to be an important component to maintain the physiological balance between bone resorption and formation. Indeed, these cells can differentiate into several other cell types such as chondrocytes, adipocytes, and osteoblasts.^3^

Regarding the differentiation process of MSCs into osteoblasts, several transcription factors are required, comprising Runt-related transcription factor 2 (RUNX2), Osterix, and β-catenin.^4^^,^^5^ The expression profile of RUNX2 is strictly regulated as it presents at a high level in preosteoblasts and then reduces to allow osteoblast maturation and differentiation into osteocytes, showing the dual role of this transcriptional factor in bone remodeling.^5^^,^^6^ Pathways implicated in the differentiation of MSCs into osteoblasts are already well described and comprise important axes such as bone morphogenetic protein (BMP), Wingless/Integrated (WNT), transforming growth factor β (TGF-β), or fibroblast growth factor (FGF) pathways, all leading to higher levels or activity of RUNX2.^4^ Interestingly, in the elderly, more prone to osteoporosis, the differentiation potential of MSCs into osteoblast decreases in favor of adipocyte differentiation.

The strict and fine-tuned regulation of the pathways implicated in bone remodeling and diseases comprises small biological molecules called microRNAs (miRNAs). miRNAs are small, highly conserved, non-coding RNAs involved in post-transcriptional regulation through the targeting of the 3′ untranslated region (UTR) of messenger RNAs (mRNAs) and degradation of the targeted mRNA or inhibition of their translation. Over the past several years, miRNAs appeared to be able to modulate bone homeostasis.^7^ The conditional skeletal gene-mediated inactivation of *Dicer*, the ribonuclease responsible for the maturation of pre-miRNA into miRNA, highlighted the role of miRNAs in both early and late stages of osteogenesis. Indeed, the ablation of *Dicer* in osteoprogenitor cells leads to bone defects in the embryo, highlighting the importance of miRNAs in bone development.^8^^,^^9^ On the contrary, deleting *Dicer* in mature osteoclasts in adult mice leads to higher bone mass.^10^ This observation suggests the existence of miRNAs that are osteo-enhancers, while others are osteo-suppressors.

Previous studies showed that specific miRNAs influence bone mineral density.^8^ Moreover, five circulating miRNAs (miR-21, miR-24, miR-23, miR-100-5p, and miR-125b) were reported as overexpressed in the plasma and at the fracture site of osteoporotic patients compared to healthy patients.^11^^,^^12^ Among these miRNAs, miR-125b, also known as miR-125b-5p, is described as able to modulate bone-related pathways.^8^^,^^13^^,^^14^ While its role in bone remodeling is not totally understood, several studies showed that miR-125b is able to reduce BMP2- and BMP4-induced differentiation of human MSCs (hMSCs).^15^ Chen and colleagues highlighted an overexpression of miR-125b in osteoporotic bone marrow-derived mesenchymal stem cells (BMSCs) compared to healthy patients.^16^ They also showed that an overexpression of miR-125b in hBMSCs inhibits their osteogenic differentiation.^16^ Moreover, this miRNA is also able to regulate osteogenesis by targeting cJun, BMP2, or BMP receptor 1b (BMPR1b).^14^ Interestingly, miR-125b is also implicated in inflammation through the downregulation of TRAF6 and A20, increasing p65, interleukin-1β (IL-1β), and IL-8 levels in HT29 cells.^17^ It is therefore not surprising that this miRNA is a focus of interest in bone diseases and remodeling.

*In vitro*, dexamethasone has been used as the gold standard model used to induce the differentiation and mineralization of hMSCs.^18^ However, the use of this glucocorticoid can be controversial because *in vivo*, it induces bone loss.^19^ In this work, we aimed to investigate the impact of miR-125b modulation in dexamethasone- and calcium-induced mineralization of hMSCs. We observed that miR-125b induced more mineralization in dexamethasone-treated cells, while it reduced it in calcium-treated cells. We therefore deepened our investigation to understand the mechanism behind these opposite effects.

## Results

### Mimicking miR-125b induced an opposite effect in dexamethasone- and calcium-treated hMSCs

**Figure 1:**
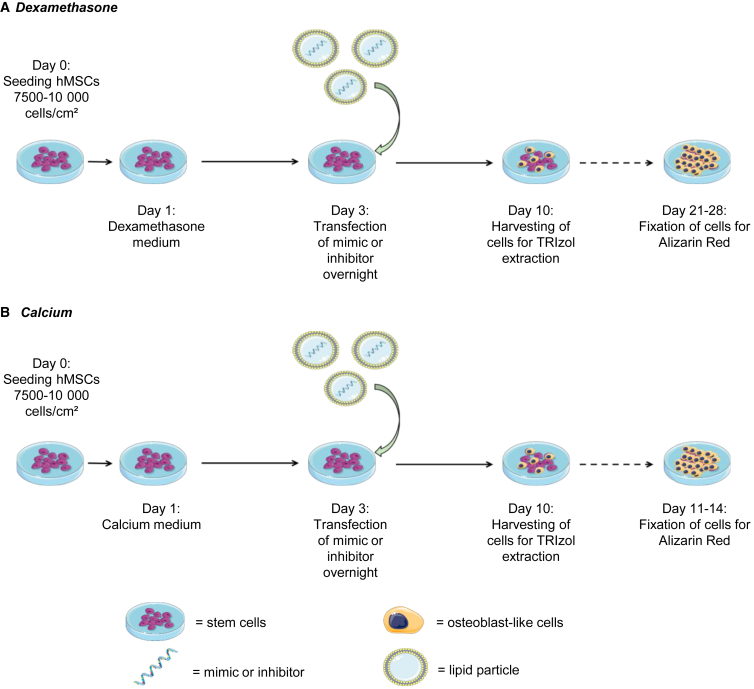
Schematic representation of dexamethasone- and calcium-induced mineralization Dexamethasone (A) or calcium chloride (B) was added to the cells one day after seeding. Transfection with mimics or negative control was performed at day 3, and cells were harvested 10 days after the beginning of dexamethasone or calcium treatment. Mineralization was assessed via alizarin red after 21–28 days for dexamethasone and 11–14 days for calcium protocol. Calcium medium, calcium chloride 8 mM and ascorbic acid; dexamethasone medium, dexamethasone 100 nM, β-glycerol phosphate, and ascorbic acid.

**Figure 2:**
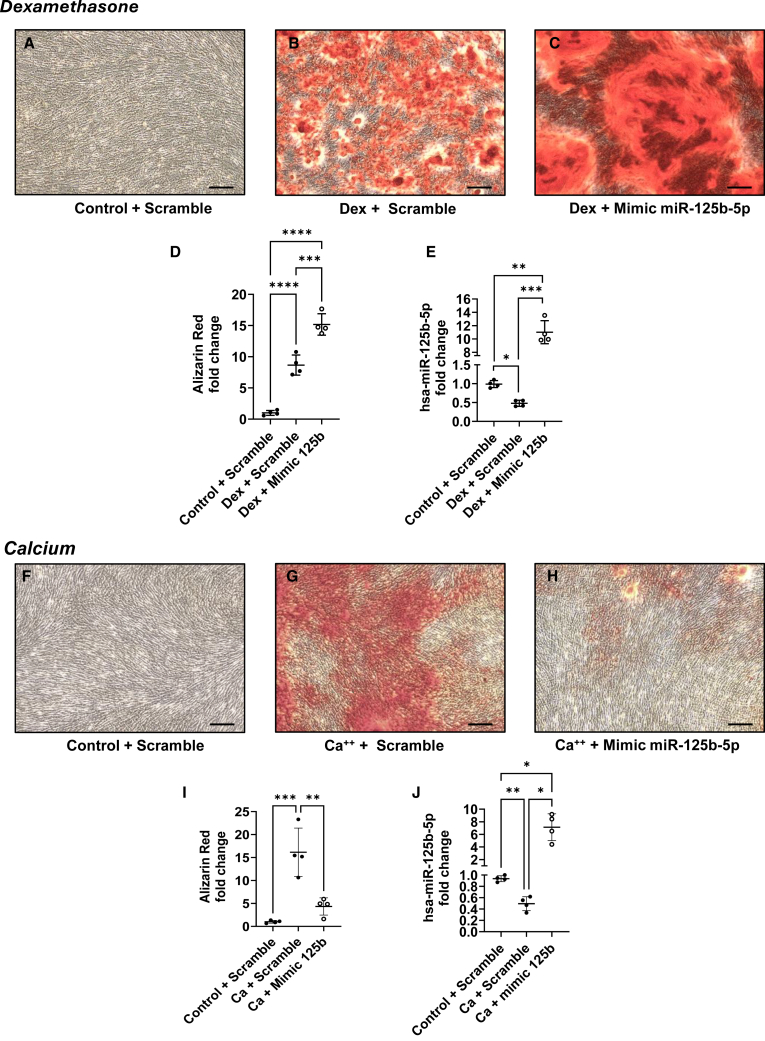
Mimicking of miR-125b induced opposite effect in dexamethasone- and calcium-treated hMSCs hMSCs were transfected with negative control or mimic and cultured in control or dexamethasone medium (top) and in control or calcium medium (bottom). Mineralization was assessed with alizarin red (A–C and F–H) and quantified with CPC (D and I) using a spectrophotometer; ∗∗*p* < 0.01; ∗∗∗*p* < 0.001; ∗∗∗∗*p* < 0.0001. miRNA-125b expression was assessed using qPCR (E and J); ∗*p* < 0.05; ∗∗*p* < 0.01; ∗∗∗*p* < 0.001 based on fold change relative to control + scramble. Scale bar, 150 μm. *N* = 4; *n* = 2. Results are expressed as mean ± SD.

Mineralization of hMSCs was induced using dexamethasone or calcium medium, and transfection was performed during the process, as described in Figures 1A and 1B. After 21 days of culture, hMSCs that were stimulated with dexamethasone medium mineralized, while control cells did not (Figures 2A and 2B). Interestingly, the transfection of miR-125b mimic in dexamethasone-stimulated cells induced higher mineralization as visualized with alizarin red (Figure 2C) and quantified by the cetylpyridinium chloride (CPC) method (Figure 2D, *p* < 0.001). Expression of miR-125b was assessed by qPCR and revealed a significant decrease of its expression in dexamethasone-treated cells compared to control, while after transfection with its mimic, we observed an 11-fold increase in miR-125b expression (Figure 2E; *p* < 0.01). In a calcium-saturated medium, after 14 days of culture, hMSCs transfected with a negative sequence (scramble) presented higher mineralization than did the control (Figures 2F and 2G). Moreover, in calcium-stimulated cells, the transfection of mimic 125b led to a decrease in mineralization (Figure 2H) as also quantified by the CPC method (Figure 2I; *p* < 0.01). Similar to what was observed for dexamethasone-containing medium, miR-125b expression was significantly downregulated in calcium-saturated medium, and the mimic transfection induced a 7.1-fold upregulation of its expression (Figure 2J; *p* < 0.05).

### BMPR2 and RUNX2 proteins presented a pattern corroborating the mineralization state

**Figure 3:**
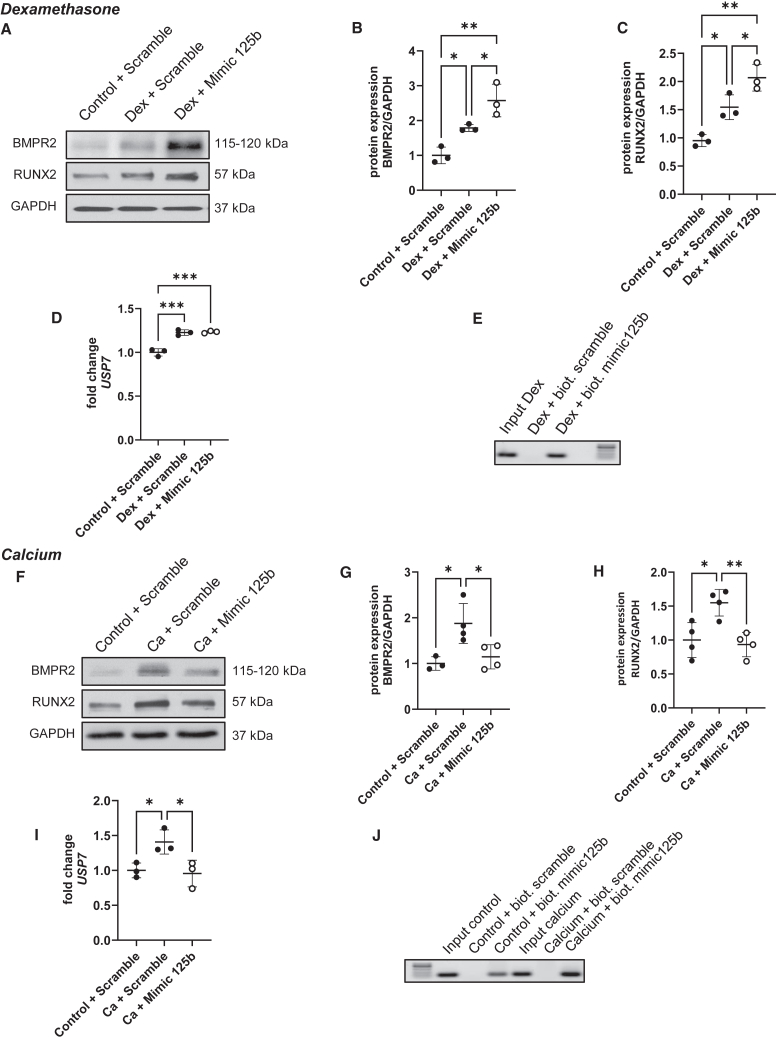
Mimicking of miR-125b induced the opposite profile of osteogenic proteins level hMSCs were transfected with negative control or mimic and cultured in control or dexamethasone medium (top) and in control or calcium medium (bottom). BMPR2 and RUNX2 levels were measured via western blotting (A and F). The quantification of protein levels was performed using ImageJ normalized to GAPDH (B, C, G, and H); ∗*p* < 0.05; ∗∗*p* < 0.01. *USP7* expression was assessed using qPCR (D and I); ∗*p* < 0.05; ∗∗∗*p* < 0.001 based on fold change relative to control + scramble. The verification of RUNX2 as a direct target of miR-125b was performed using a pull-down assay with biotinylated mimic-125b and negative control combined with PCR and agarose gel (E and J). *N* = 3–4; *n* = 2. Results are expressed as mean ± SD.

After 7 days, BMPR2 and RUNX2 levels were significantly increased in scrambled-transfected hMSCs after dexamethasone stimulation compared to control cells (Figures 3A–3C). Similar to what we observed with alizarin red staining, their level was even higher in the dexamethasone-treated group transfected with the miR-125b mimic. Moreover, *USP7* expression, a stabilizer of RUNX2, was increased with dexamethasone and remained upregulated when the miR-125b mimic was transfected (Figure 3D; *p* < 0.001). Interestingly, we showed that RUNX2 is a direct target of miR-125b in dexamethasone-treated cells (Figure 3E). hMSCs transfected with scrambled mimic and cultured in the presence of calcium also presented increased levels of BMPR2 and RUNX2 compared to control cells. However, when transfected in calcium-treated cells, the miR-125b mimic induced a significant decrease in these two proteins (Figures 3F–3H). While *USP7* expression is increased in calcium-treated cells, it returns to control levels in cells transfected with the mimic (Figure 3I; *p* < 0.05). As observed for dexamethasone-treated cells, RUNX2 was observed to be a direct target of miR-125b in calcium-treated cells (Figure 3J).

### Mimicking miR-125b differentially regulated miR-199a/214 cluster in dexamethasone- and calcium-treated hMSCs

**Figure 4:**
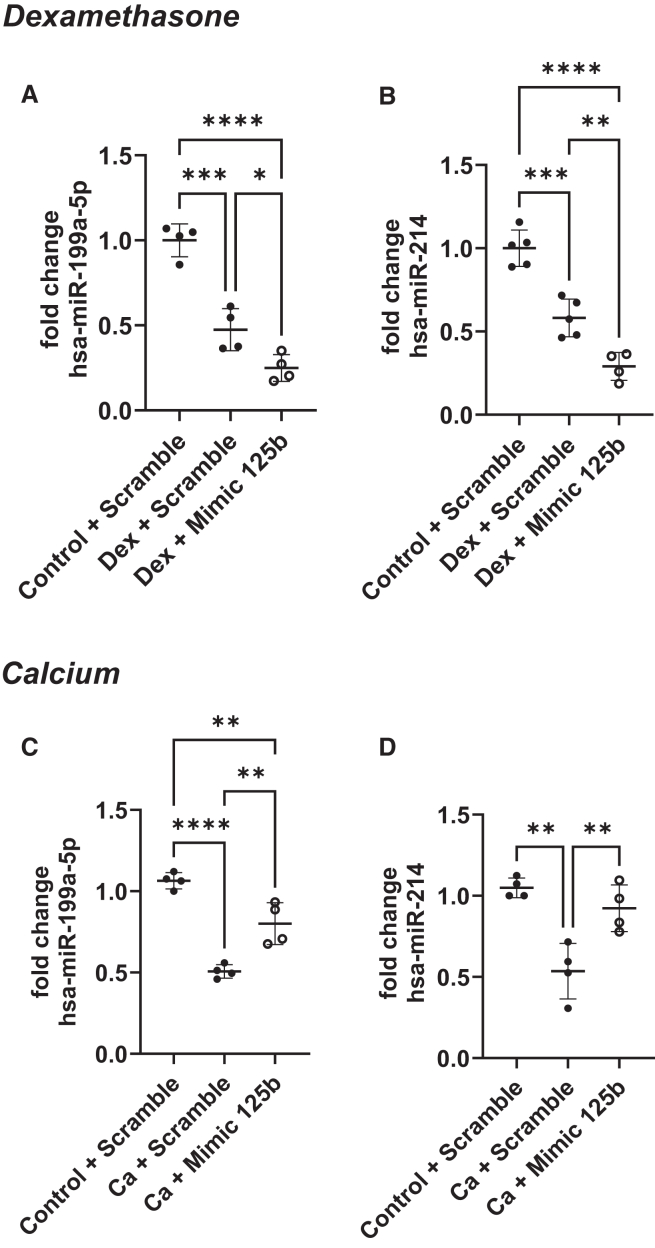
Mimicking miR-125b differentially regulated miR-199a/214 cluster in dexamethasone- and calcium-treated hMSCs hMSCs were transfected with negative control or mimic and cultured in control or dexamethasone medium (top) and in control or calcium medium (bottom). miR-199a-5p and miR-214 expression was measured using qPCR. ∗*p* < 0.05; ∗∗*p* < 0.01; ∗∗∗*p* < 0.001; ∗∗∗∗*p* < 0.0001 based on fold change relative to control + scramble. *N* = 4–5; *n* = 2. Results are expressed as mean ± SD.

The expression of miR-199a-5p and miR-214 was measured in hMSCs stimulated with dexamethasone or calcium for 7 days and transfected with scramble or miR-125b mimic. We observed that both dexamethasone (Figures 4A and 4B) and calcium (Figures 4C and 4D) treatments induced a significant downregulation of miR-199a-5p and miR-214 in hMSCs. However, transfection of miR-125b mimic in dexamethasone-stimulated cells emphasized this downregulation (Figures 4A and 4B; *p* < 0.05 and *p* < 0.01) while it counteracted it in calcium-stimulated cells. Indeed, after transfection with miR-125b mimic, calcium-treated cells presented a higher expression of the miR-199a/214 cluster compared to calcium-treated cells transfected with the scrambled-miR (Figures 4C and 4D; *p* < 0.01). Interestingly, in dexamethasone-treated cells, the downregulation of miR-199a/214 cluster is associated with a significant increase in caveolin-1, a well-known target of miR-199a-5p (Figure S1A). However, the upregulation of the miR199a/214 cluster in calcium-treated cells is associated with a reduction in caveolin-1 expression (Figure S1B).

### Signal transducer and activator of transcription 3 and tumor protein p53 were direct targets of miR-125b in hMSCs but behaved differently in dexamethasone- and calcium-treated cells transfected with mimic

**Figure 5:**
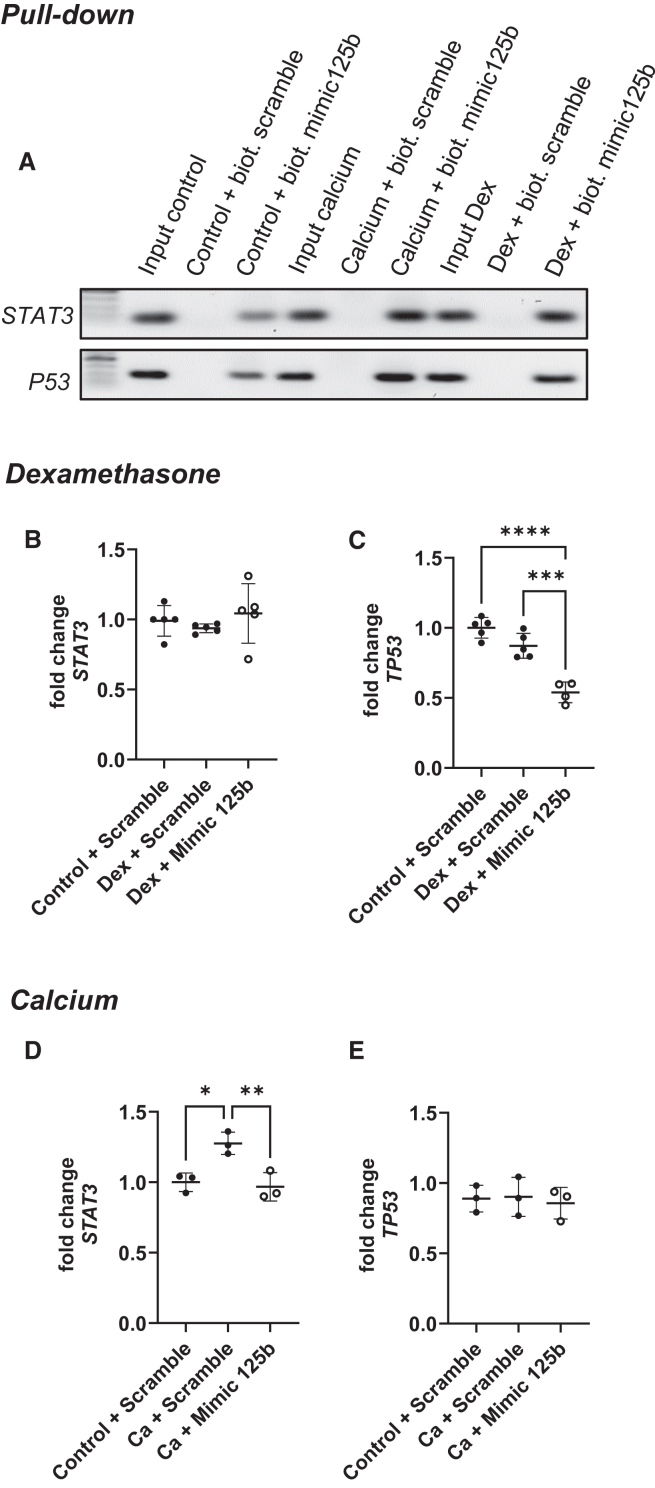
The direct targets STAT3 and p53 reacted differently to miR-125b in dexamethasone and calcium medium The verification of STAT3 and TP53 as a direct target of miR-125b was performed using a pull-down assay with biotinylated mimic-125b and negative control combined with PCR and agarose gel (A). hMSCs were transfected with negative control or mimic and cultured in control or dexamethasone medium (center) and in control or calcium medium (bottom). STAT3 (B and D) and p53 (C and E) expression was measured using qPCR. ∗*p* < 0.05; ∗∗*p* < 0.01; ∗∗∗*p* < 0.001; ∗∗∗∗*p* < 0.0001 based on fold change relative to control + scramble. *N* = 3–5; *n* = 2. Results are expressed as mean ± SD.

After performing a pull-down assay using biotinylated miR-125b mimic or negative control, we observed that signal transducer and activator of transcription 3 (*STAT3)* and *p53* were direct targets of miR-125b (Figure 5A). However, their expression did not vary in the same way in transfected dexamethasone- or calcium-stimulated cells. Indeed, while transfection of miR-125b in dexamethasone-treated cells did not induce any modification of *STAT3* expression (Figure 5B), *p53* expression decreased compared to other conditions (Figure 5C; *p* < 0.001). However, calcium treatment induced a significant overexpression of *STAT3*, and the concomitant transfection of miR-125b counteracted it (Figure 5D; *p* < 0.01), while *p53* expression was not modified (Figure 5E).

### Rescuing miR-214 expression in dexamethasone-treated hMSCs transfected with miR-125b mimic abrogated mineralization

**Figure 6:**
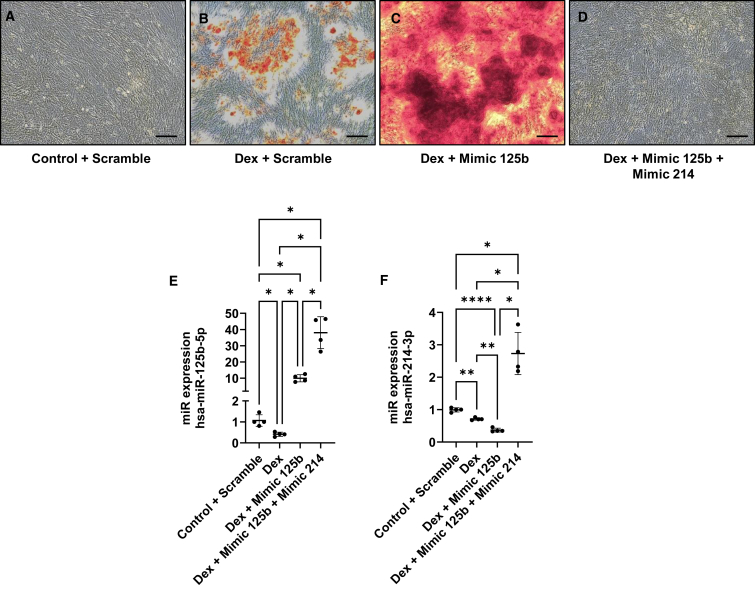
Rescuing miR-214 in dexamethasone-treated hMSCs transfected with miR-125b abrogated mineralization hMSCs were transfected with negative control or mimic and cultured in control or dexamethasone medium. Mineralization was assessed with alizarin red (A–D). miRNA-125b and miR-214 expression was assessed using qPCR (E and F). ∗*p* < 0.05; ∗∗*p* < 0.01; ∗∗∗∗*p* < 0.0001 based on fold change relative to control + scramble. *N* = 4; *n* = 2. Scale bar, 150 μm. Results are expressed as mean ± SD.

To understand whether miR-214 could be the culprit of the effect observed in dexamethasone-treated hMSCs, we co-transfected miR-125b and miR-214 mimics. We observed that, as previously reported, dexamethasone induced mineralization of hMSCs compared to control medium (Figures 6A and 6B). However, while the transfection with miR-125b mimic enhanced the mineralization induced by dexamethasone (Figure 6C), the cells transfected with miR-125b and miR-214 mimics did not show mineralization per se (Figure 6D). Measurement of miR-125b and miR-214 expressions confirmed the efficiency of the transfection (Figures 6E and 6F).

## Discussion

Musculoskeletal disorders are considered a burdensome health problem, with osteoporotic fractures as a major contributor. Osteoporosis is a silent disease until a fracture occurs, and, for now, the available treatments cause several adverse effects. Osteoporosis is characterized by an imbalance between bone formation and resorption, leading to more fragile bone.^1^ The mechanisms behind bone remodeling are partially known and require several pathways as BMP, FGF, TGF, and Wnt.^4^ However, fine-tuned regulation of these pathways is not yet well studied and understood.

In this work, we tried to elucidate the role of miR-125b in bone metabolism. miR-125b is a member of a five-miRNAs signature found in the blood and fracture site of osteoporotic patients.^11^^,^^12^ Here, we showed that the modulation of miR-125b in hMSCs induced different effects on mineralization, depending on the *in vitro* model used. We observed an increase in mineralization in dexamethasone-treated hMSCs with miR-125b. However, we were surprised to find that the concomitant mimicking of miR-125b in those cells induced a higher mineralization than the dexamethasone alone. In fact, several studies reported miR-125b to be able to reduce osteogenic differentiation of MSCs. Mizuno et al. showed that overexpression of miR-125b induces the inhibition of proliferation of ST2 cells, followed by the suppression of BMP4-induced osteogenesis.^20^ Huang et al. reported that miR-125b expression is decreased during osteogenic differentiation of C3H10T1/2 cells and that its overexpression reduces mineralization through the downregulation of the Cbfβ gene.^15^ In BMSCs, the overexpression of miR-125b leads to the suppression of osteogenic differentiation, probably by targeting Osterix.^16^ Wang et al. showed that in hMSCs, miR-125b can reduce osteogenic differentiation via a downregulation of BMPR1b.^14^ *In vivo*, the role of miR-125b in bone is more shaded. While some studies show that miR-125b overexpression blocks osteoclastogenesis and promotes higher bone mass,^21^ other works design miR-125b as a culprit of osteoporotic phenotype.^13^

To further investigate the results obtained, we wanted to reproduce it in another osteogenic *in vitro* model consisting of calcium-saturated medium using calcium chloride. The use of extracellular calcium ions to induce mineralization of MSCs is already well developed using both calcium-coated surface or calcium ion-saturated medium.^22^^,^^23^^,^^24^ In this model, we observed that culturing the cells in calcium medium increased the mineralization of hMSCs, as observed for dexamethasone. However, in this condition, the mimicking of miR-125b drastically reduced this effect. Interestingly, the difference observed in mineralization between dexamethasone and calcium-treated cells was associated with an opposite pattern of osteogenic proteins such as BMPR2 and RUNX2. Indeed, while both treatments induced an increase in the two proteins, miR-125b mimic emphasized the upregulation in dexamethasone-treated cells and counteracted it in calcium-treated cells. While BMPR2 is a well-known target of miR-125b in musculoskeletal disorders,^13^^,^^25^ RUNX2 is always described as an indirect target.^26^ In this work, we showed that RUNX2 is a direct target of miR-125b in hMSCs. Moreover, we also observed a differential expression of USP7, known to deubiquitinate RUNX2 and avoid its degradation by the proteasome. Kim et al. showed that knockdown of USP7 in MSCs reduces RUNX2 and osteopontin levels, while another study of theirs highlighted the importance of deubiquitination of RUNX2 by USP7 for bone formation.^27^^,^^28^ These results indicate that in our model, miR-125b might be able to impact RUNX2 by regulating both its expression and stabilization.

To better understand the reason for the opposite effect obtained in dexamethasone- and calcium-treated hMSCs transfected with miR-125b mimic, we performed a literature search and *in silico* studies using databases such as TargetScan, miRanda, and Tools4miR, as well as alignment prediction software such as miRmap. We highlighted two potential targets: STAT3 and p53. In their study about osteosarcoma, Liu et al. showed that miR-125b can directly target STAT3, thereby reducing the proliferation and migration of the cancerous cells.^29^ Moreover, miR-125b is described as a negative regulator of p53 in neuroblastoma cells, where it reduces their apoptosis.^30^ More recently, a study showed that increased levels of miR-125b in the human breast cancer cell line MCF7 induced a decrease in p53, and this was associated with DNA damage.^31^ In the hMSCs used in this study, we confirmed, via pull-down assay, that STAT3 and p53 are targets of miR-125b in both dexamethasone- and calcium-treated cells. However, their expression showed a different pattern in both treatments. Interestingly, calcium is known to induce STAT3 expression, while dexamethasone alone is not.^32^^,^^33^ This corroborates what we observed in hMSCs. Opposite that from our observations, dexamethasone was previously reported to increase p53 in MC3T3-E1 osteoblastic cells.^34^ However, this has been investigated mostly at the protein level and not the RNA level, as investigated in the present study.

The identification of STAT3 and p53 as direct targets of miR-125b combined in *in silico* studies, prediction alignment, transcription factor prediction (TransMiR), and literature search led us to a specific cluster of miRNAs, the miR-199a/214 cluster. STAT3 is reported to be a modulator of miR-199a and miR-214 expression in cardiac and thyroid tissues.^35^^,^^36^ p53 is also associated with miR-199a-5p and miR-214 expression during renal fibrosis, and it has been identified as a transcription factor of these two miRNAs.^37^^,^^38^ With this in mind, we measured the expression of miR-199a-5p and miR-214 and showed that it was also differentially regulated in dexamethasone- and calcium-treated cells. Both treatments induced a downregulation of the two miRNAs but mimicking miR-125b in dexamethasone reduced their expression even more. Conversely, overexpression of miR-125b in calcium-treated cells restored the level of miR-199a-5p and miR-214 in hMSCs. Moreover, the rescue of miR-214 in dexamethasone-treated cells transfected with miR-125b mimic drastically reduced the mineralization of the cells. miR-214 is reported to be a biomarker of osteoporosis, and it is known that upon production by osteoclasts, this miRNA can inhibit osteoblast differentiation in ovariectomized mice.^39^ Also, Yuan et al. showed that shear stress due to physical exercise downregulates the expression of miR-214 in tibial bone and increased expression of osteogenic markers.^40^ While the effect of miR-199a-5p on bone formation is not clear, its downregulation in dexamethasone-treated cells was associated with an increase of caveolin-1 expression, one of its targets.^41^ Interestingly, BMPR2 is localized in caveolae, and the downregulation of caveolin-1 leads to reduced localization of BMPR2 at the membrane.^42^ Therefore, the upregulation of caveolin-1 observed in dexamethasone-treated cells could contribute to the increased mineralization by increasing the amount of BMPR2 at the basal membrane. However, the downregulation of caveolin-1 due to miR-125b transfection in calcium-treated cells could participate in the decreased mineralization observed. The suspected pathways implicated in this work are shown in Figure S2.

Dexamethasone is often used to induce the differentiation of stem cells into osteogenic lineages.^43^ However, its effect on osteogenic differentiation is controversial. Prolonged use of dexamethasone *in vivo* induces osteoporosis and increases the risk of fracture.^19^ Moreover, Li et al. showed that BMSCs isolated from dexamethasone-treated mice differentiate into adipocytes instead of osteoblasts.^44^ In 2004, a study showed that dexamethasone can inhibit the mineralization of osteoblasts in a dose- and time-dependent manner.^45^ The controversy around dexamethasone has not been elucidated. Recently, dexamethasone was shown to be able to induce osteogenic differentiation of MSCs through the downregulation of SOX9 and not through the upregulation of RUNX2 as is usually thought.^46^ This was also correlated with an upregulation of peroxisome proliferator-activated receptor-γ, inducing the appearance of adipocyte-like cells and showing that dexamethasone is probably not the best *in vitro* model to induce osteogenesis.^46^ Our present work aligns with this statement. Results obtained when transfecting dexamethasone-treated cells with miR-125b mimic align with what is observed in the literature. Furthermore, our obtained results support the statement that the miRNA expression profile is dependent on the specific *in vitro* mineralization model used. We are aware that the timeline of mineralization differs between the two treatments, but we do not suspect any implication of this in the effect of miR-125b on the mineralization, as cells are transfected in the early beginning of the process for both protocols. As dexamethasone medium also contains β-glycerophosphate (BGP), which is absent from Ca^2+^-saturated medium, this could also influence the effect of miR-125b overexpression on mineralization. However, as Wen and colleagues showed, miR-125b conserves its anti-mineralization effect in high phosphate environments.^47^ Moreover, we induced differentiation in medium containing only BGP and ascorbic acid phosphate (ASAP) without dexamethasone and observed that cells are not reaching mineralization within 28 days. Interestingly, the overexpression of miR-125b in these cells is not improving the appearance of mineral deposition (data not shown). These results make us think that the differential effect observed comes from the dexamethasone and not from the BGP.

In conclusion, in this work, we showed that dexamethasone and calcium *in vitro* mineralization models react differently to the overexpression of miR-125b. We showed that while the targets of miR-125b are the same in the two treatments, their regulation by the miR is different and impacts the downstream cascade, including the miR-199a/214 cluster. It is interesting to highlight that the overexpression of miR-125b induces a modulation of other miRNAs. Furthermore, the treatment-dependent difference reported in this work emphasizes the importance of *in vivo* or clinical study design. Treatments such as dexamethasone are useful to treat certain pathologies but remain highly stressful, and as observed in this study, they may deeply impact the cellular response, even at the epigenetic level. To better understand the mechanisms behind the differential response observed in the two treatments, a more detailed study must be performed focusing on miR-199a-5p and miR-214. This work also draws attention to miR-125b, miR-199a-5p, and miR-214 as potential therapeutic targets for bone diseases.

## Materials and methods

### Isolation and culture of hMSCs

Primary hMSCs were isolated from human BM aspirate provided by the Department of Trauma Surgery from the Maastricht University Medical Center+ hospital during routine orthopedic surgical procedures. Samples from 5 different donors (age range: 17–66 years old, mean age 53 years old, 3 males, 2 females) were used for this work. Informed consent was obtained from each donor, as well as approval from the ethical committee (ethical number METC 15-4-274), following the Declaration of Helsinki guidelines. Patients presenting with autoimmune diseases were excluded from the study. To isolate cells, BM aspirates were placed in Dulbecco’s modified Eagle’s medium (Hams F12 [1:1]; Thermo Fisher Scientific, Waltham, MA), supplemented with 10% fetal calf serum (Thermo Fisher Scientific), 2.5 μg/mL amphotericin B (Thermo Fisher Scientific), 100 μg/mL penicillin/streptomycin (Thermo Fisher Scientific), and 7.5% sodium hydrogen carbonate (Merck, Darmstadt, Germany), and buffered with HEPES buffer (Thermo Fisher Scientific). After centrifugation for 5 min at 1,750 × *g*, the cell pellet was sorted using a Percoll gradient (Amersham Biosciences, Little Chalfont, UK; d = 1.131 g/mL) for 15 min at 1,750 × *g*. After one wash with culture medium, the cell layer was cultivated in a humidified atmosphere at 37°C and 5% CO_2_ in standard cell culture flasks. After the adhesion of cells, the supernatant containing dead cells was discarded. For daily culture, hMSCs were maintained in α-minimum essential medium (α-MEM; Thermo Fisher Scientific), supplemented with 10% fetal bovine serum (FBS; Thermo Fisher Scientific) without antibiotics. The medium was refreshed every 2 days, and cells were passaged using 0.05% trypsin-EDTA (Thermo Fisher Scientific) when confluency reached 80%–90%. For experiments, cells were not used after reaching passage 6. Before any experiment, the multipotency of each donor was validated by inducing their differentiation into adipocytes, chondrocytes, or osteoblasts.

### Dexamethasone and calcium stimulation

hMSCs were seeded at a density of 7,500 cells/cm^2^ in a 6-well plate and maintained in α-MEM with 10% FBS without antibiotics. After attachment, cells were cultured in dexamethasone medium consisting of α-MEM with 10% FBS, 10 mM BGP (Sigma, St. Louis, MO), 0.02 mM ASAP, and 100 nM dexamethasone or calcium-saturated medium consisting of α-MEM containing 10% FBS, 8 mM calcium chloride, and 0.02 mM ASAP (Figure 1). Control cells were maintained in α-MEM supplemented with 10% FBS. After 24 h, the cells were transfected overnight (ON), as described below. Subsequently, the cell culture medium was replaced with control medium, dexamethasone medium, or calcium-saturated medium until the end of the culture. Media changes were performed every 2 days. No antibiotics were added during the whole culture, including transfection and mineralization. For immunoblotting and qPCRs, cells were harvested 7 days after transfection. The alizarin red staining to assess the mineralization of the cells was performed 14 and 21 days after the induction of calcium or dexamethasone stimulation, respectively.

### Transfection of hMSCs

Transfection of hMSCs was performed 24 h after the beginning of differentiation in both dexamethasone- and calcium-treated cells. Cells were transfected ON with 25 nM scrambled miR (negative control; Qiagen, Hilden, Germany) or 25 nM miR-125b mimic (Qiagen) or a combination of 25 nM miR-125b mimic and 10 nM of miR-214 mimic (Qiagen) using Lipofectamine 3000 (Invitrogen, Waltham, MA) in Opti-MEM and diluted in α-MEM with 10% FBS, according to the manufacturer’s protocol. Medium was refreshed with basal, dexamethasone, or calcium-saturated medium and renewed every 2 days until the end of the culture.

### Extraction of mRNAs and miRNAs

mRNAs and miRNAs were extracted using TRIzol (Invitrogen) as advised by the manufacturer’s protocol. Briefly, cells were harvested in TRIzol, and chloroform (200 μL/mL TRIzol; Sigma) was added. Samples were mixed and incubated at room temperature (RT) for 15 min. After centrifugation for 20 min at 4°C, the aqueous phase was transferred in new tubes and isopropanol (500 μL/mL TRIzol; Fisher Scientific, Hampton, NH) was added, incubated for 15 min at RT, and centrifuged for 20 min at 4°C. Isopropanol was then removed, and the pellet was washed twice with a cold 70% ethanol solution. RNA concentration was measured using a Biodrop device (Fisher Scientific).

### miRNA reverse transcription and qPCR

Following the manufacturer’s instructions, the first-strand cDNA synthesis was performed using the miRCURY LNA RT Kit (Qiagen). Briefly, 40 ng RNA was loaded in the reaction, and a mix containing RT buffer, water, and RT enzyme was added to a final volume of 10 μL. Samples were incubated at 42°C for 60 min, followed by an inactivation reaction of 5 min at 95°C. Samples were then diluted, and 3 μL were loaded in duplicate in a 96-well qPCR plate. The miRCURY SYBR Green Master Mix (Qiagen) along with PCR primers for hsa-miR-125b, hsa-miR-199a-5p, hsa-miR-214, or U6 (Qiagen; Table S1) were added, and a PCR run was performed in a CFX96 machine (Bio-Rad, Hercules, CA) following the manufacturer’s protocol. The samples underwent an initial heat activation at 95°C for 2 min, followed by a two-step cycling consisting of a first step at 95°C for 10 s and a second step at 56°C for 60 s. The two-step cycling was repeated 40 times before melting curve analysis was performed. Quantification was expressed as fold induction of the miRNA of interest compared with U6 following the 2^−ΔΔCT^ formula. Conditions were normalized to the cells cultured in basal medium and transfected with the negative sequence (scramble).

### mRNA reverse transcription and qPCR

The first-strand cDNA synthesis was performed using the iScript kit (Bio-Rad). A total of 500 ng RNA was used in the reverse transcription reaction, and a mix containing RNAse-free water (Qiagen), RT enzyme, and RT buffer was added to a final volume of 20 μL. Samples were incubated at 25°C for 5 min for a priming step, followed by an incubation at 46°C for 20 min and an inactivation step at 95°C for 1 min. cDNA was diluted at a concentration of 5 ng/μL, and 2 μL was loaded in duplicate in a 96-well qPCR plate with the reaction mix comprising 5 μL SYBR Green Master Mix (Bio-Rad), 2 μL water, and 1 μL primer mix containing forward and reverse primers for *STAT3* and *P53* (Table S1). The real-time PCR CFX96 was programmed to perform 40 cycles of 10 s of denaturation at 95°C and 60 s of combined annealing and extension at 60°C. Genes of interest were normalized to the housekeeper gene *GAPDH* (Table S1). Quantification was expressed as fold induction of the gene of interest compared with GAPDH (glyceraldehyde 3-phosphate dehydrogenase) following the 2^−ΔΔCT^ formula. Conditions were normalized to the cells cultured in basal medium and transfected with the negative sequence (scramble).

### Western blot

Proteins were extracted using TRIzol following the manufacturer’s protocol and quantified using the Pierce bicinchoninic acid assay (Thermo Fisher Scientific). Western blot was performed on an 8%–10% acrylamide gel, and 15 μg proteins were loaded per well. Electrophoresis was performed for 15 min at 90 V in a running buffer consisting of 25 mM Tris, 192 mM glycine, 0.1% SDS, pH 8.3 following dilution to 1× with water and then increased to 120 V for 60 min. After separation, the proteins were transferred onto a nitrocellulose membrane with a constant amperage of 350 mA for 90 min in a transfer buffer (50 mM Tris-base, 40 mM glycine, 1.5 mM SDS). Membranes were blocked in Tris-buffered saline (TBS)-5% bovine serum albumin (BSA)-0.1% Tween 20 for 60 min at RT. Primary antibodies used for immunodetection were diluted in TBS-5% BSA-0.1% Tween and incubated ON at 4°C. The primary antibodies were anti-SMAD4 (1/1,000; Cell Signaling Technology, Danvers, MA), anti-RUNX2 (1/1,000; Thermo Fisher Scientific), anti-BMPR2 (1/1,000; Novus Biologicals, Centennial, CO), and anti-GAPDH (1/10,000; Cell Signaling Technology). Membranes were rinsed with TBS-0.1% Tween 20 and incubated with horseradish peroxidase-conjugated secondary antibodies diluted in TBS-5% BSA-0.1% Tween 20 for 60 min at RT. Development was performed using Bio-Rad Clarity substrate (Bio-Rad) and CL-Xposure film (Thermo Fisher Scientific). Protein bands were quantified by densitometry using ImageJ software (National Institutes of Health). Levels of proteins of interest were normalized to GAPDH levels.

### Alizarin red staining and quantification

To assess the mineralization of cells after dexamethasone and calcium stimulation, an alizarin red staining was performed. Cells were fixed in 4% paraformaldehyde solution diluted in phosphate-buffered saline (PBS) for 15 min at RT and rinsed twice in PBS and once in distilled water. A 1% alizarin red solution diluted in water and adjusted to pH 4.1–4.2 was added to cells and incubated for 30 min at RT under agitation. Cells were rinsed several times with distilled water, and imaging was performed on an inverted Nikon Ti-S/L100 microscope, equipped with a Nikon DS-Ri2 camera using a CFI Plan Apochromat K 20× objective (numerical aperture: 0.75, working distance: 1.0). Images were analyzed using NIS-Elements software (version 5.30.06, Nikon, Tokyo, Japan). Quantification of the staining was performed using 10% CPC diluted in sodium phosphate buffer. CPC was incubated ON at RT under gentle agitation. CPC solution was transferred in a 96-well plate, and absorbance was measured at 562 nm using a ClarioStar spectrophotometer (BMG Labtech, Champigny-sur-Marne, France). Concentration was determined using a standard curve of alizarin red diluted in CPC solution.

### Pull-down assay

hMSCs were transfected with a biotinylated mimic of miR-125b or biotinylated negative control sequence. After 7 and 14 days cultured in a 6-well plate in basal medium, calcium-saturated medium, or osteogenic medium, cells were harvested by scraping one well in 200 μL lysis buffer (20 mM Tris-base, pH 7.5, 100 mM KCl, and 5 mM MgCl_2_) added with 0.05% Tween 20, 50 U RNAse OUT (Invitrogen), and proteinase inhibitor cocktail (PIC, Sigma). Before pull-down, 150 μL Dynabeads M-270 streptavidin (Invitrogen) was washed three times with lysis buffer. Beads were blocked for 2 h under agitation at 4°C in 800 μL lysis buffer added with 0.05% Tween 20, 50 U RNAse OUT, PIC, 200 yeast tRNA (1 mg/mL; Invitrogen), and 1% BSA. After two washes, 150 μL harvested cells, coming from one 6-well plate, were incubated with the beads for 4 h at 4°C under agitation. Beads were delicately washed three times in 500 μL lysis buffer and resuspended in TRIzol LS (Invitrogen) and extracted for RNAs following the manufacturer’s protocol.

### PCR and gel electrophoresis

After RNA extraction of the pull-down samples, RNA pellets were resuspended in 10 μL RNAse-free water (Qiagen). First-strand cDNA synthesis was done using 8 μL RNA and the iScript kit (Bio-Rad), as previously described. Classical PCR was performed with a GoTaq Green enzyme (Promega, Madison, WI) in a three-step protocol. The thermocycler was programmed to perform 35 cycles of 30 s of denaturation at 95°C, 60 s of annealing at 60°C, and 60 s of extension at 72°C. After amplification, the samples were run on a 1% agarose gel for 90 min at 120 V. Imaging was performed using Chemidoc technology (Bio-Rad). The genes of interest were *RUNX2*, *STAT3*, and *p53* (Table S1).

### Statistical analyses

Statistical analyses were performed on GraphPad Prism version 10.0.2. All data were expressed as the mean ± standard deviation. Experiments were performed in duplicate (n = 2) in at least three independent donors (*N* = 3). Normality was checked using the Shapiro-Wilk normality test. Significant differences (∗*p* < 0.05; ∗∗*p* < 0.01; ∗∗∗*p* < 0.001; ∗∗∗∗*p* < 0.0001) between groups were assessed using a one-way ANOVA, followed by a Tukey-Kramer post hoc ANOVA test. Nonparametric data were analyzed using a Mann-Whitney test. A Grubb’s test was performed to detect potential outliers.

## Data and code availability

Upon publication, all data associated with this paper will be made publicly available or upon request from the corresponding author (Virginie Joris).

## Acknowledgments

This work was supported by the 10.13039/100020979Province of Limburg (The Netherlands), Limburg Invests in its Knowledge Economy (LINK). We would like to thank Maria Paula Marks, Maria Eischen-Loges, and Steven Vermeulen from the MERLN Institute for Technology-Inspired Regenerative Medicine for their technical help.

## Author contributions

V.J. designed the work, performed the data acquisition and data analysis, and wrote and reviewed the manuscript. E.R.B. contributed to the design of the work and review of the manuscript. M.v.G. conceived and designed the project, analyzed the data, wrote and reviewed the manuscript, and acquired the funding.

## Declaration of interests

The authors declare no competing interests.

## Supplemental information

Document S1. Figures S1 and S2 and Table S1

Document S2. Article plus supplemental information

## References

[1] Li H., Xiao Z., Quarles L.D., Li W. (2021). Osteoporosis: Mechanism, Molecular Target and Current Status on Drug Development. Curr. Med. Chem..

[2] Cummings S.R., San Martin J., McClung M.R., Siris E.S., Eastell R., Reid I.R., Delmas P., Zoog H.B., Austin M., Wang A. (2009). Denosumab for prevention of fractures in postmenopausal women with osteoporosis. N. Engl. J. Med..

[3] Infante A., Rodríguez C.I. (2018). Osteogenesis and aging: lessons from mesenchymal stem cells. Stem Cell Res. Ther..

[4] Ponzetti M., Rucci N. (2021). Osteoblast Differentiation and Signaling: Established Concepts and Emerging Topics. Int. J. Mol. Sci..

[5] Nakashima K., de Crombrugghe B. (2003). Transcriptional mechanisms in osteoblast differentiation and bone formation. Trends Genet..

[6] Maruyama Z., Yoshida C.A., Furuichi T., Amizuka N., Ito M., Fukuyama R., Miyazaki T., Kitaura H., Nakamura K., Fujita T. (2007). Runx2 determines bone maturity and turnover rate in postnatal bone development and is involved in bone loss in estrogen deficiency. Dev. Dynam..

[7] Seeliger C., Balmayor E.R., van Griensven M. (2016). miRNAs Related to Skeletal Diseases. Stem Cell. Dev..

[8] Lian J.B., Stein G.S., van Wijnen A.J., Stein J.L., Hassan M.Q., Gaur T., Zhang Y. (2012). MicroRNA control of bone formation and homeostasis. Nat. Rev. Endocrinol..

[9] Gaur T., Hussain S., Mudhasani R., Parulkar I., Colby J.L., Frederick D., Kream B.E., van Wijnen A.J., Stein J.L., Stein G.S. (2010). Dicer inactivation in osteoprogenitor cells compromises fetal survival and bone formation, while excision in differentiated osteoblasts increases bone mass in the adult mouse. Dev. Biol..

[10] Mizoguchi F., Izu Y., Hayata T., Hemmi H., Nakashima K., Nakamura T., Kato S., Miyasaka N., Ezura Y., Noda M. (2010). Osteoclast-specific Dicer gene deficiency suppresses osteoclastic bone resorption. J. Cell. Biochem..

[11] Kelch S., Balmayor E.R., Seeliger C., Vester H., Kirschke J.S., van Griensven M. (2017). miRNAs in bone tissue correlate to bone mineral density and circulating miRNAs are gender independent in osteoporotic patients. Sci. Rep..

[12] Seeliger C., Karpinski K., Haug A.T., Vester H., Schmitt A., Bauer J.S., van Griensven M. (2014). Five freely circulating miRNAs and bone tissue miRNAs are associated with osteoporotic fractures. J. Bone Miner. Res..

[13] Wang G., Zhang L., Yan C., Wang F., Zhang Y. (2021). Overexpression of miR125b Promotes Osteoporosis Through miR-125b-TRAF6 Pathway in Postmenopausal Ovariectomized Rats. Diabetes Metab. Syndr. Obes..

[14] Wang H., Xie Z., Hou T., Li Z., Huang K., Gong J., Zhou W., Tang K., Xu J., Dong S. (2017). MiR-125b Regulates the Osteogenic Differentiation of Human Mesenchymal Stem Cells by Targeting BMPR1b. Cell. Physiol. Biochem..

[15] Huang K., Fu J., Zhou W., Li W., Dong S., Yu S., Hu Z., Wang H., Xie Z. (2014). MicroRNA-125b regulates osteogenic differentiation of mesenchymal stem cells by targeting Cbfbeta in vitro. Biochimie.

[16] Chen S., Yang L., Jie Q., Lin Y.S., Meng G.L., Fan J.Z., Zhang J.K., Fan J., Luo Z.J., Liu J. (2014). MicroRNA-125b suppresses the proliferation and osteogenic differentiation of human bone marrow-derived mesenchymal stem cells. Mol. Med. Rep..

[17] Valmiki S., Ahuja V., Puri N., Paul J. (2019). miR-125b and miR-223 Contribute to Inflammation by Targeting the Key Molecules of NFkappaB Pathway. Front. Med..

[18] Langenbach F., Handschel J. (2013). Effects of dexamethasone, ascorbic acid and beta-glycerophosphate on the osteogenic differentiation of stem cells in vitro. Stem Cell Res. Ther..

[19] Weinstein R.S. (2012). Glucocorticoid-induced osteoporosis and osteonecrosis. Endocrinol Metab. Clin. N. Am..

[20] Mizuno Y., Yagi K., Tokuzawa Y., Kanesaki-Yatsuka Y., Suda T., Katagiri T., Fukuda T., Maruyama M., Okuda A., Amemiya T. (2008). miR-125b inhibits osteoblastic differentiation by down-regulation of cell proliferation. Biochem. Biophys. Res. Commun..

[21] Ito S., Minamizaki T., Kohno S., Sotomaru Y., Kitaura Y., Ohba S., Sugiyama T., Aubin J.E., Tanimoto K., Yoshiko Y. (2021). Overexpression of miR-125b in Osteoblasts Improves Age-Related Changes in Bone Mass and Quality through Suppression of Osteoclast Formation. Int. J. Mol. Sci..

[22] Aquino-Martinez R., Artigas N., Gamez B., Rosa J.L., Ventura F. (2017). Extracellular calcium promotes bone formation from bone marrow mesenchymal stem cells by amplifying the effects of BMP-2 on SMAD signalling. PLoS One.

[23] Shih Y.R.V., Hwang Y., Phadke A., Kang H., Hwang N.S., Caro E.J., Nguyen S., Siu M., Theodorakis E.A., Gianneschi N.C. (2014). Calcium phosphate-bearing matrices induce osteogenic differentiation of stem cells through adenosine signaling. Proc. Natl. Acad. Sci. USA.

[24] Lee M.N., Hwang H.S., Oh S.H., Roshanzadeh A., Kim J.W., Song J.H., Kim E.S., Koh J.T. (2018). Elevated extracellular calcium ions promote proliferation and migration of mesenchymal stem cells via increasing osteopontin expression. Exp. Mol. Med..

[25] Xiao Y., Yan X., Yang Y., Ma X. (2019). Downregulation of long noncoding RNA HOTAIRM1 variant 1 contributes to osteoarthritis via regulating miR-125b/BMPR2 axis and activating JNK/MAPK/ERK pathway. Biomed. Pharmacother..

[26] Frohlich L.F. (2019). Micrornas at the Interface between Osteogenesis and Angiogenesis as Targets for Bone Regeneration. Cells.

[27] Kim J.M., Yang Y.S., Park K.H., Ge X., Xu R., Li N., Song M., Chun H., Bok S., Charles J.F. (2020). A RUNX2 stabilization pathway mediates physiologic and pathologic bone formation. Nat. Commun..

[28] Kim Y.J., Park K.H., Lee K.M., Chun Y.M., Lee J.W. (2022). Deubiquitinating Enzyme USP7 Is Required for Self-Renewal and Multipotency of Human Bone Marrow-Derived Mesenchymal Stromal Cells. Int. J. Mol. Sci..

[29] Liu L.H., Li H., Li J.P., Zhong H., Zhang H.C., Chen J., Xiao T. (2011). miR-125b suppresses the proliferation and migration of osteosarcoma cells through down-regulation of STAT3. Biochem. Biophys. Res. Commun..

[30] Le M.T.N., Teh C., Shyh-Chang N., Xie H., Zhou B., Korzh V., Lodish H.F., Lim B. (2009). MicroRNA-125b is a novel negative regulator of p53. Genes Dev..

[31] Goswami B., Ahuja D., Pastré D., Ray P.S. (2023). p53 and HuR combinatorially control the biphasic dynamics of microRNA-125b in response to genotoxic stress. Commun. Biol..

[32] Jeong J., Lee J., Talaia G., Kim W., Song J., Hong J., Yoo K., Gonzalez D., Athonvarangkul D., Shin J. (2024). Intracellular calcium links milk stasis to lysosome-dependent cell death during early mammary gland involution. Cell Mol Life Sci.

[33] Huang P., Sun R., Xu C., Jiang Z., Zuo M., Li Y., Liu R., Gong P., Han Y., Fang J. (2024). Glucocorticoid activates STAT3 and NF-kappaB synergistically with inflammatory cytokines to enhance the anti-inflammatory factor TSG6 expression in mesenchymal stem/stromal cells. Cell Death Dis..

[34] Zhou P., Xia D., Wang Y., Lv H., Wang Z., Xing M., Zhao Q., Xu S. (2019). Matrine derivate MASM protects murine MC3T3-E1 osteoblastic cells against dexamethasone-induced apoptosis via the regulation of USP14/p53. Artif. Cells, Nanomed. Biotechnol..

[35] Craps J., Joris V., Baldeschi L., Daumerie C., Camboni A., Buemi A., Lengelé B., Behets C., Boschi A., Mourad M. (2021). miR-199a Downregulation as a Driver of the NOX4/HIF-1alpha/VEGF-A Pathway in Thyroid and Orbital Adipose Tissues from Graves' Patients. Int. J. Mol. Sci..

[36] Haghikia A., Missol-Kolka E., Tsikas D., Venturini L., Brundiers S., Castoldi M., Muckenthaler M.U., Eder M., Stapel B., Thum T. (2011). Signal transducer and activator of transcription 3-mediated regulation of miR-199a-5p links cardiomyocyte and endothelial cell function in the heart: a key role for ubiquitin-conjugating enzymes. Eur. Heart J..

[37] Ma Z., Li L., Livingston M.J., Zhang D., Mi Q., Zhang M., Ding H.F., Huo Y., Mei C., Dong Z. (2020). p53/microRNA-214/ULK1 axis impairs renal tubular autophagy in diabetic kidney disease. J. Clin. Invest..

[38] Yang R., Xu X., Li H., Chen J., Xiang X., Dong Z., Zhang D. (2017). p53 induces miR199a-3p to suppress SOCS7 for STAT3 activation and renal fibrosis in UUO. Sci. Rep..

[39] Li D., Liu J., Guo B., Liang C., Dang L., Lu C., He X., Cheung H.Y.S., Xu L., Lu C. (2016). Osteoclast-derived exosomal miR-214-3p inhibits osteoblastic bone formation. Nat. Commun..

[40] Yuan Y., Guo J., Zhang L., Tong X., Zhang S., Zhou X., Zhang M., Chen X., Lei L., Li H. (2019). MiR-214 Attenuates the Osteogenic Effects of Mechanical Loading on Osteoblasts. Int. J. Sports Med..

[41] Jin H.Q., Jiang W.F., Zheng X.T., Li L., Fang Y., Yang Y., Hu X.W., Chu L.S. (2023). MiR-199a-5p enhances neuronal differentiation of neural stem cells and promotes neurogenesis by targeting Cav-1 after cerebral ischemia. CNS Neurosci. Ther..

[42] Tomita S., Nakanishi N., Ogata T., Higuchi Y., Sakamoto A., Tsuji Y., Suga T., Matoba S. (2024). The Cavin-1/Caveolin-1 interaction attenuates BMP/Smad signaling in pulmonary hypertension by interfering with BMPR2/Caveolin-1 binding. Commun. Biol..

[43] Ter Brugge P.J., Jansen J.A. (2002). In vitro osteogenic differentiation of rat bone marrow cells subcultured with and without dexamethasone. Tissue Eng..

[44] Li J., Zhang N., Huang X., Xu J., Fernandes J.C., Dai K., Zhang X. (2013). Dexamethasone shifts bone marrow stromal cells from osteoblasts to adipocytes by C/EBPalpha promoter methylation. Cell Death Dis..

[45] Leclerc N., Luppen C.A., Ho V.V., Nagpal S., Hacia J.G., Smith E., Frenkel B. (2004). Gene expression profiling of glucocorticoid-inhibited osteoblasts. J. Mol. Endocrinol..

[46] Della Bella E., Buetti-Dinh A., Licandro G., Ahmad P., Basoli V., Alini M., Stoddart M.J. (2021). Dexamethasone Induces Changes in Osteogenic Differentiation of Human Mesenchymal Stromal Cells via SOX9 and PPARG, but Not RUNX2. Int. J. Mol. Sci..

[47] Wen P., Cao H., Fang L., Ye H., Zhou Y., Jiang L., Su W., Xu H., He W., Dai C., Yang J. (2014). miR-125b/Ets1 axis regulates transdifferentiation and calcification of vascular smooth muscle cells in a high-phosphate environment. Exp. Cell Res..

