# Supplementary material for: miR-125b differentially impacts mineralization in dexamethasone and calcium-treated human mesenchymal stem cells
Source: Mol Ther Nucleic Acids. 2025 Jan 3;36(1):102446. doi: 10.1016/j.omtn.2024.102446 (PMC11787018; doi:10.1016/j.omtn.2024.102446)
Supplement: Document S1. Figures S1 and S2 and Table S1 [file mmc1.pdf]

OMTN, Volume *36*

Supplemental information

miR-125b differentially impacts mineralization in dexamethasone and calcium-treated human mesenchymal stem cells

Virginie Joris, Elizabeth R. Balmayor, and Martijn van Griensven

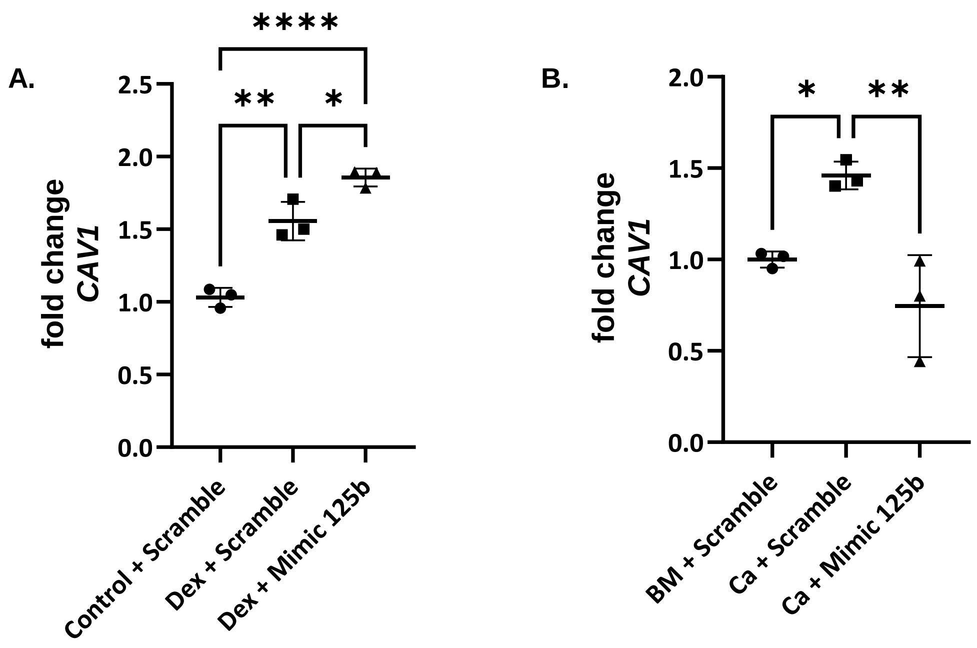

Figure S1: **Caveolin-1 is differentially regulated in dexamethasone and calcium-treated cells transfected with miR-125b.** hMSCs were transfected with scramble or mimic and cultured in control, dexamethasone (A) or calcium (B) medium. CAV1 expression was measured using qPCR. *$p < 0.05$; **$p < 0.01$; ****$p < 0.0001$ based on fold change relative to control + scramble.

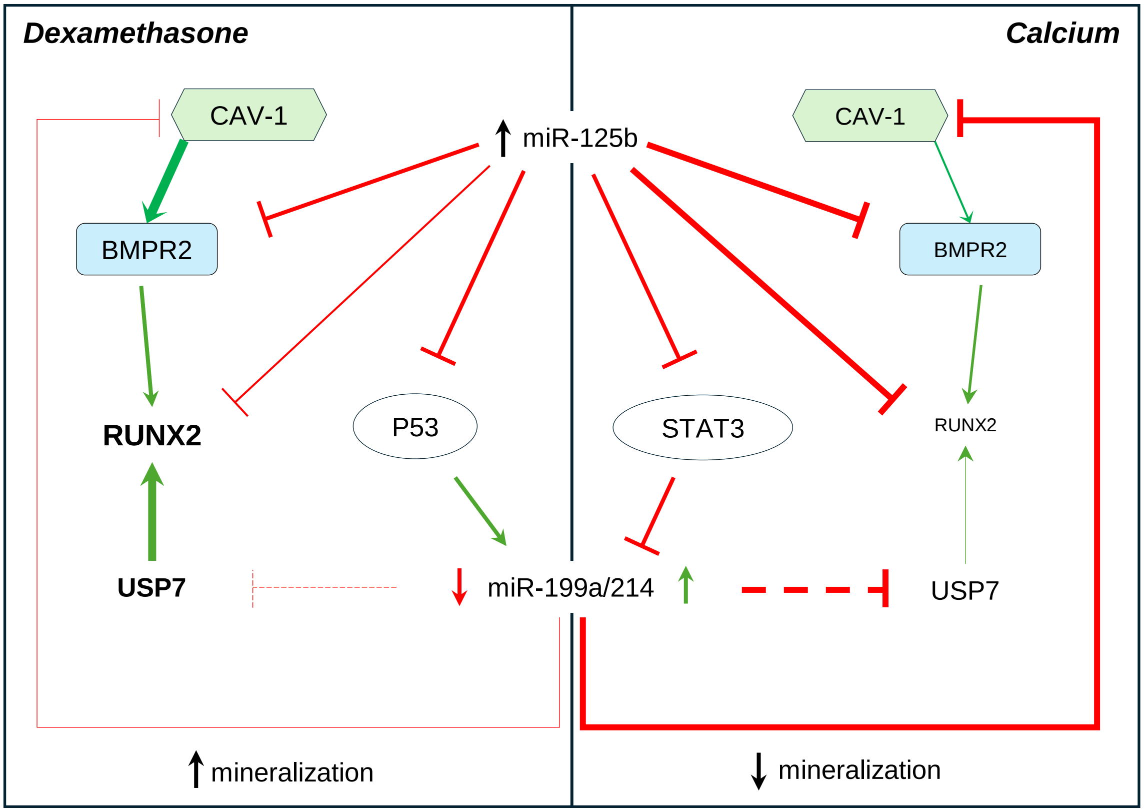

Figure S2: **Overexpression of miR-125b induces opposite effect of mineralization in dexamethasone- and calcium-treated cells.** The left panel shows that overexpression of miR-125b in dexamethasone-treated cells decreases p53 leading to lower expression of miR-199a/214 cluster. This relieves the inhibition of USP7 and BMPR2 (via CAV-1) leading to higher level of RUNX2 translated by higher mineralization. At the opposite, the right panels shows that the overexpression of miR-125b in calcium-treated cells reduces STAT3 expression leading to an increase of miR-199a/214 cluster. This strengthens the inhibition of USP7 and BMPR2 and leads to lower level of RUNX2 translated by lower mineralization. The dotted lines are based on prediction softwares, the plain lines are proven interaction through literature or pull-down assay.

Table S1: **Primers used for qPCR.** Primers for miRNAs are designed and purchased by Qiagen

Primers	Sequences forward (F) and reverse (R) 5' to 3'
GAPDH	F: CTGGGCTACACTGAGCACC<br>R: AAGTGGTCGTTGAGGGCAATG
USP7	F: GGACATGGAGGATGACACCA<br>R: TCACTCAGTCTGCTGAAGCG
STAT3	F: AGCAGTTTCTTCAGAGCAGGT<br>R: CACAATCCGGGCAATCTCCA
P53	F: CCCTGCCCTCAACAAGATGT<br>R: CTCCGTCATGTGCTGTGACT
RUNX2	F: TCAACGATCTGAGATTTGTGGG<br>R: GGGGAGGATTTGTGAAGACGG
Hsa-miR-125b-5p	Qiagen Cat. Number: 339306<br>GeneGlobe ID: YP00205713
Hsa-miR-199a-5p	Qiagen Cat. Number: 339306<br>GeneGlobe ID: YP00204494
Hsa-miR-214-3p	Qiagen Cat. number: 339306<br>GeneGlobe ID: YP00204510
U6	Qiagen Cat. number: 339306<br>GeneGlobe ID: YP02119464
